# Expression Analysis of Nitrogen Metabolism-Related Genes Reveals Differences in Adaptation to Low-Nitrogen Stress between Two Different Barley Cultivars at Seedling Stage

**DOI:** 10.1155/2018/8152860

**Published:** 2018-06-20

**Authors:** Zhiwei Chen, Chenghong Liu, Yifei Wang, Ting He, Runhong Gao, Hongwei Xu, Guimei Guo, Yingbo Li, Longhua Zhou, Ruiju Lu, Jianhua Huang

**Affiliations:** ^1^Biotechnology Research Institute of Shanghai Academy of Agricultural Sciences, 2901 Beidi Road, Minhang District, Shanghai 201106, China; ^2^Shanghai Key Laboratory of Agricultural Genetics and Breeding, 2901 Beidi Road, Minhang District, Shanghai 201106, China

## Abstract

The excess use of nitrogen fertilizers causes many problems, including higher costs of crop production, lower nitrogen use efficiency, and environmental damage. Crop breeding for low-nitrogen tolerance, especially molecular breeding, has become the major route to solving these issues. Therefore, in crops such as barley (*Hordeum vulgare* L.), it is crucial to understand the mechanisms of low-nitrogen tolerance at the molecule level. In the present study, two barley cultivars, BI-04 (tolerant to low nitrogen) and BI-45 (sensitive to low nitrogen), were used for gene expression analysis under low-nitrogen stress, including 10 genes related to primary nitrogen metabolism. The results showed that the expressions of *HvNIA2* (nitrite reductase), *HvGS2* (chloroplastic glutamine synthetase), and *HvGLU2* (ferredoxin-dependent glutamate synthase) were only induced in shoots of BI-04 under low-nitrogen stress, *HvGLU2* was also only induced in roots of BI-04, and *HvGS2* showed a rapid response to low-nitrogen stress in the roots of BI-04. The expression of *HvASN1* (asparagine synthetase) was reduced in both cultivars, but it showed a lower reduction in the shoots of BI-04. In addition, gene expression and regulation differences in the shoots and roots were also compared between the barley cultivars. Taken together, the results indicated that the four above-mentioned genes might play important roles in low-nitrogen tolerance in barley.

## 1. Introduction

Nitrogen, one of the essential elements for crop growth and development, is a primary driver of crop production. Thus, many new crop varieties with high yields, dependent on high-nitrogen fertilizer input, were introduced into crop production in the 20th century according to the preferences of farmers and breeders [1]. However, the increased use of nitrogen fertilizer caused a number of problems, such as high input costs for crop production, a decrease in nitrogen use efficiency, nitrogen fertilizer loss, and environmental pollution [2]. As a result, there is now a consensus among plant and environmental scientists that it is important to balance the benefits of nitrogen application, mainly increased yield, against its disadvantages, and to minimize negative impacts by decreasing nitrogen fertilizer input and environmental pollution, while maintaining yields. Therefore, increasing nitrogen use efficiency or developing crops with the ability to tolerate low-nitrogen are important targets for future crop breeding [2]. Achieving these targets will require a comprehensive understanding of nitrogen metabolism under low-nitrogen condition, particularly the expression of genes involved in the adaptation to, or tolerance of, low-nitrogen stress.

Nitrogen physiology is complicated, comprising processes such as acquisition, assimilation, transportation, remobilization, and the metabolism of nitrogen-containing compounds [2–4]. The most commonly used external chemical nitrogen sources are nitrate and ammonium. Nitrate is taken up by low- and/or high-affinity nitrate transport systems (NRT1 and NRT2), while ammonium is taken up by ammonium transporters (AMT). The high-affinity nitrate transporters play important roles under nitrogen starvation or low-nitrogen stress [5]. The absorbed nitrate is firstly reduced to nitrite by nitrate reductase (NR) and then reduced to ammonium by nitrite reductase (NiR). Ammonium is assimilated into amino acids in a process that is catalyzed mainly by the GS-GOGAT (glutamine synthetase and glutamate synthase) pathway. Another important enzyme is asparagine synthetase (AS), which catalyzes the transfer of glutamine to asparagine. Asparagine is a major molecular nitrogen for nitrogen transportation in many plant species because it has a relatively high nitrogen : carbon ratio [6], and the translocation of nitrogen within plants is also very important for plant growth and seed development [7].

Barley (*Hordeum vulgare* L.) is a model plant for cereal research, as well as being an important crop, and many of its nitrogen metabolism-related genes have been cloned [8–18]. There have been reports comparing nitrogen metabolism-related gene expression in different plant genotypes with different responses to low-nitrogen stress [19–21]. Although there was a recent study concerning transcriptome analysis under low-nitrogen stress in the roots of two different barley genotypes [22], the different definition of low-nitrogen tolerance and only using root tissue for the transcriptome analysis might not be enough to fully understand their responses to low-nitrogen stress.

In the present study, we first determined a suitable degree of low-nitrogen stress to obtain significant differences in the phenotypes between two barley cultivars with different responses to low nitrogen. We compared the expression patterns of genes related to nitrogen metabolism in the shoots and roots of the two barley cultivars. The aim was to acquire information concerning the differential regulation of nitrogen metabolism-related genes between low-nitrogen-tolerant and low-nitrogen-sensitive barley cultivars under low-nitrogen treatment and to reveal their roles in adaptation to low-nitrogen stress.

## 2. Materials and Methods

### 2.1. Plant Growth and Low-Nitrogen Treatments

Barley BI-04 is a relatively low-nitrogen-tolerant cultivar, while barley BI-45 is a relatively low-nitrogen-sensitive cultivar [23, 24]. Seeds of the two cultivars were sterilized by immersion in 1% NaClO and germinated in an incubator at 25°C for one week. Seedlings were cultured in nutrient solution comprising 1.43 mM NH_4_NO_3_, 0.32 mM NaH_2_PO_4_ · 2H_2_O, 0.51 mM K_2_SO_4_, 1.00 mM CaCl_2_, 3.36 mM MgSO4, 9.47 *μ*M MnSO_4_·H_2_O, 0.08 *μ*M Na_2_MoO_4_·H_2_O, 19.42 *μ*M H_3_BO_3_, 0.15 *μ*M ZnSO4·7H_2_O, 0.16 *μ*M CuSO_4_·5H_2_O, and 61.24 *μ*M iron citrate (mainly according to [25]). Seedlings were transferred into nutrient solution with the NH_4_NO_3_ concentration reduced to 19.21 mg·L^−1^ (0.24 mM) at the fourth leaf stage. The pH was maintained at 6.2 ± 0.3. Plants in hydroponic growth boxes were kept in an artificial incubator with a 16/8 h (light/dark) cycle at 20°C ± 2°C and 70% relative humidity. Shoots and roots were harvested separately at 0, 1, 24, and 48 h after low-nitrogen treatment, frozen in liquid nitrogen, and kept at −80°C. There were three biological replicates for each sample. For biomass investigation, BI-04 and BI-45 plants were constantly cultured for another one week from the fourth leaf stage; one group of plants was grown with a normal nitrogen supply (1.43 mM NH_4_NO_3_), and the other group was grown under low-nitrogen stress (0.24 mM NH_4_NO_3_). The plants were then harvested, and shoots and roots were collected separately. There were 20 biological replicates of each variety under each treatment.

For biomass measurements, all shoots and roots of BI-04 and BI-45 were incubated at 105°C for 30 min and dried at 80°C for about 2 days until their weight remained constant weight, as determined using an electronic analytical balance.

### 2.2. RNA Extraction and cDNA Synthesis

Total RNA from shoot and root samples was isolated by using TRIzol (Invitrogen, Carlsbad, CA, USA) and treated with RQ1 RNase-Free DNase (Promega, Madison, WI, USA) to degrade any contaminating genomic DNA. cDNA was synthesized using SuperScript III Reverse Transcriptase (Invitrogen, USA) and checked for purity by using polymerase chain reaction (PCR) amplification with primers CATCAAGCTCAAGGACGACA and GCCTTGTCCTTGTCAGTGAA, which anneal to sites flanking an intron within the *HvGAPDH* gene. The presence of contaminating genomic DNA would lead to amplification of a 229 bp product in addition to the 150 bp product amplified from the cDNA [26].

### 2.3. Quantitative Real-Time PCR (qPCR)

Primers were designed using primer 3 (http://primer3.wi.mit.edu/) (Table 1). PCR reactions were performed in 96-well plates on a 7500 Real-Time PCR System (Applied Biosystems, Foster, CA, USA) using SYBR Select Master Mix (Applied Biosystems), according to the manufacturer's instructions. The reactions for sets of three biological replicate samples per time point were separated across three plates, thus forming statistical blocks for subsequent data analysis. Reactions contained 10 *μ*L 2x mix, 0.6 *μ*L of each primer (1 *μ*M), and 100 ng of cDNA template in a final volume of 20 *μ*L. The same thermal profile was used for all PCR reactions: 50°C for 2 min, 95°C for 2 min, and 45 cycles of 95°C for 15 s and 60°C for 1 min. Data collection was carried out during the 60°C step. Dissociation/melting curves were constructed after cycle 45.

### 2.4. Statistical Analysis

Biomass comparisons and gene expression comparisons between the shoots and the roots were analyzed statistically using a *t*-test in Excel 2007 software.

The efficiency of the PCR was estimated using the LinReg PCR program [27]. The cycle threshold (Ct) value was obtained using 7500 software v2.0.5 (Applied Biosystems), and the Ct and efficiency values were then used to calculate the relative quantity (RQ) and the normalized relative quantity (NRQ) of a target gene's expression with respect to two reference genes, *HvActin* and *HvGAPDH*. The NRQ was calculated using the following formula:

(1) $$\begin{array}{c} NRQ=\frac{E_{target}^{-Ct,target}}{\sqrt{E_{HvActin}^{-Ct,HvActin}\cdot E_{HvGAPDH}^{-Ct,HvGAPDH}}}. \end{array}$$

Statistical analysis of the NRQ data was also according to Chen et al. [26].

## 3. Results

### 3.1. Effects of Low-Nitrogen Treatment on Plant Growth and Biomass in the Two Barley Cultivars

**Figure 1:**
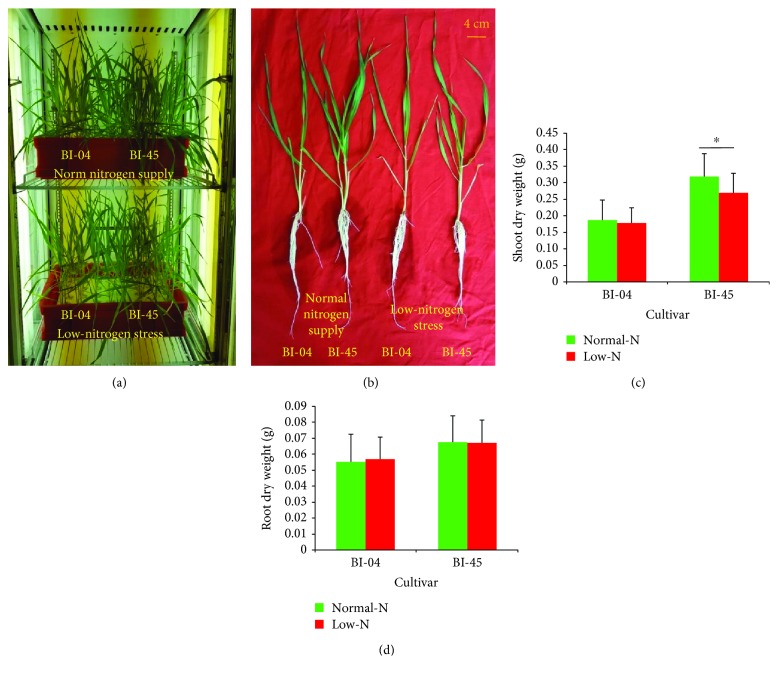
Plant growth and performance of BI-04 (low-N tolerant) and BI-45 (low-N sensitive) under normal nitrogen supply and low-nitrogen stress. (a) Plant growth and treatment in an artificial climate incubator. (b) Plant performances under different nitrogen conditions. (c) Shoot dry weight (mean and SD, *n* = 20) under different nitrogen conditions. (d) Root dry weight (mean and SD, *n* = 20) under different nitrogen conditions. Significance levels of differences between normal nitrogen supply and low-nitrogen stress were estimated according to the two-tailed *t*-test method (^∗^ *P* < 0.05).

BI-04 was considered a low-nitrogen-tolerant barley cultivar, while BI-45 is a low-nitrogen-sensitive [23, 24]. In this study, the growth of BI-04 and BI-45 seemed to be suppressed, accompanied by chlorosis, under low-nitrogen stress, and the restriction was more serious in BI-45 (Figures 1(a) and 1(b)). Comparing the biomass, there was no significant difference in shoot dry weight of BI-04 between normal nitrogen supply and low-nitrogen stress, while there was a significant difference in BI-45 (*P* < 0.05), and there were no significant differences in root dry weight of the two barley cultivars between normal nitrogen supply and low-nitrogen stress (Figures 1(c) and 1(d)). The results indicated that the responses to low-nitrogen stress were different between BI-04 and BI-45 and that BI-04 was more tolerant to low-nitrogen stress than BI-45. The results also suggested that the responses to low-nitrogen stress were different between shoots and roots and the restriction of barley growth caused by low-nitrogen stress first happened in the shoots.

### 3.2. Identification of Genes Involved in Nitrogen Metabolism in Barley

The aim of this study was to analyze the expression levels of genes involved in nitrogen metabolism under low-nitrogen stress at the seedling stage in two barley cultivars, including genes encoding NRT2, NR, NiR, GS, GOGAT, and AS. All gene sequences were downloaded directly from the NCBI database, and the accession numbers are given below in parentheses.

The genes that were chosen for analysis included two high-affinity nitrate transporter genes, *HvNRT2.2* (gb|U34290.1) and *HvNRT2.3* (gb|AF091115.1) ([17]; Vidmar et al. [18]). Expression of these genes was studied only in the roots of the barley cultivars. Two other nitrate transporter genes, *HvNRT3.1* (gb|AY253448.1) and *HvNRT3.3* (gb|AY253450.1), which function with NRT2 as a two-component high-affinity nitrate uptake system [16], were also selected and assessed in the roots and shoots of the barley cultivars. NRT3 is much smaller than NRT2 and has fewer transmembrane domains [16].

One nitrate reductase gene, *HvNIA2* (gb| X57845.1), which encodes an NADH-specific nitrate reductase [13], was studied in both shoots and roots in the barley cultivars, as was a putative nitrite reductase-related gene, *HvNiR1* (gb|S78730.1) [9]. Barley contains another nitrate reductase gene, *HvNIA1* (gb| X60173.1), which encodes a NAD(P)H-bispecific nitrate reductase; however, this gene is normally expressed at very low levels, especially when *HvNIA2* is expressed ([11]; Sue et al. [15]).

Two glutamine synthetase genes, *HvGS1_1* (gb| X69087.1) which encodes cytoplasmic glutamine synthetase [10, 28] and *HvGS2* (gb| X53580.1) which encodes chloroplastic glutamine synthetase [14, 28], were included in the study and analyzed in the shoots and roots in the barley cultivars. One glutamate synthase gene, *HvGLU2* (gb|S58774.1), which encodes ferredoxin-dependent glutamate synthase [8], was also studied in the shoots and roots. There are two main types of GOGAT in higher plants, Fd-GOGAT and NADH-GOGAT, and the Fd-GOGAT activity is dominant in plants [29].

One asparagine synthetase gene, *HvASN1* (gb|AF307145.1), was studied in the shoots and roots of the barley cultivars [12, 28]. Two genes that encode asparagine synthetase were studied; however, the expression of *HvASN2* (gb|AY193714.1) was found to be very low and unstable, especially in roots, which is consistent with the report of Moller et al. [12], so it was not used for further expression analysis.

### 3.3. Expression Analyses in Shoots of Two Barley Cultivars

**Figure 2:**
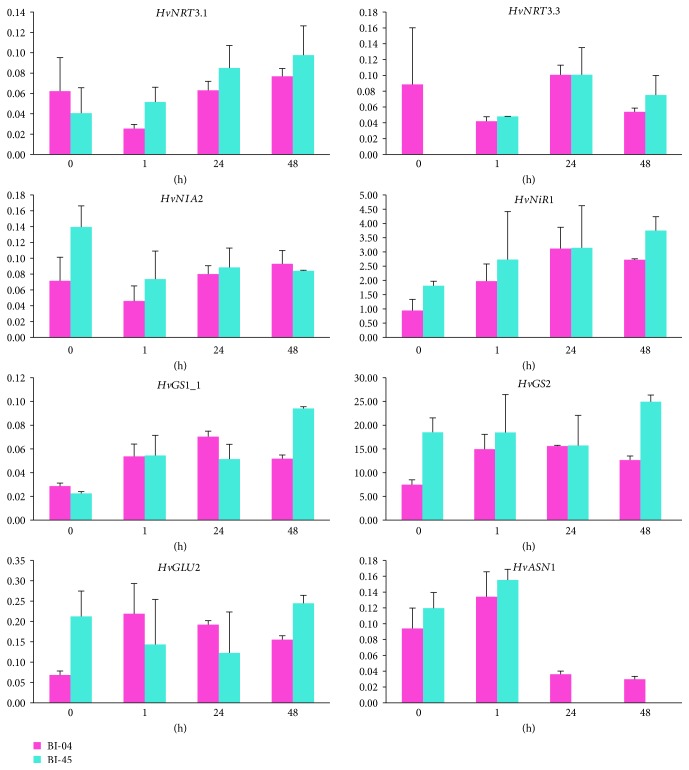
Differential expression of genes related to nitrogen metabolism in shoots of the two barley cultivars. Shoots were sampled at 0 h, 1 h, 24 h, and 48 h after low-nitrogen stress. Expression is represented as the normalized relative quantity (NRQ) of a target gene's expression with respect to the two reference genes: *HvActin* and *HvGAPDH*. Means and standard errors are shown from the analysis of three biological replicates.

The expression levels of *HvNRT3.1*, *HvNRT3.3*, *HvNIA2*, *HvNiR1*, *HvGS1_1*, *HvGS2*, *HvGLU2*, and *HvASN1* were assessed in the shoots of the two barley cultivars under low-nitrogen stress (Figure 2). Analysis of variance (ANOVA) showed that the expression of *HvGS2* in the shoots was significantly different between the barley cultivars (*P* < 0.05), the expression level of *HvGS1_1* and *HvASN1* showed significant differences among different time points (*P* < 0.05), and there was a significant interaction in the *HvGS1_1* between barley cultivars and time points (*P* < 0.05) (see Supplementary Table S1).

In the multiple comparison analysis of gene expression, *HvNRT3.1*, *HvNRT3.3*, and *HvNIA2* showed no significant changes in response to low-nitrogen stress in the shoots of BI-04, while *HvNRT3.1*, *HvNIA2*, *HvNiR1*, *HvGS2*, and *HvGLU2* showed no significant changes in BI-45 (see Supplementary Table S5). These results indicated that gene regulation was more sensitive in BI-04 than in BI-45 in the shoots under low-nitrogen stress. For BI-04, *HvGS1_1*, *HvGS2*, and *HvGLU2* were significantly induced after 1 h of low-nitrogen treatment (*P* < 0.05), and *HvNiR1* was significantly induced after 24 h of low-nitrogen treatment (*P* < 0.05), while *HvASN1* was significantly reduced after 48 h of low-nitrogen treatment (*P* < 0.05). In BI-45, *HvNRT3.3* and *HvGS1_1* were significantly induced at 1 h after low-nitrogen treatment (*P* < 0.05), and *HvNRT3.3* was hardly detectable under normal nitrogen supply, while *HvASN1* was reduced and became undetectable from 24 h after low-nitrogen treatment.

Comparing the two barley cultivars, *HvNiR1*, *HvGS2*, and *HvGLU2* were only induced in BI-04. This suggested that these three genes might play important roles in the low-nitrogen tolerance of BI-04. In addition, *HvASN1* was reduced in both barley cultivars; however, the expression of *HvASN1* in BI-45 almost disappeared from 24 h after low-nitrogen treatment. The results suggested that the lower expression of *HvASN1* in BI-04 from 24 h after low-nitrogen stress might have a positive effect on low-nitrogen tolerance.

### 3.4. Expression Analyses in the Roots of Two Barley Cultivars

**Figure 3:**
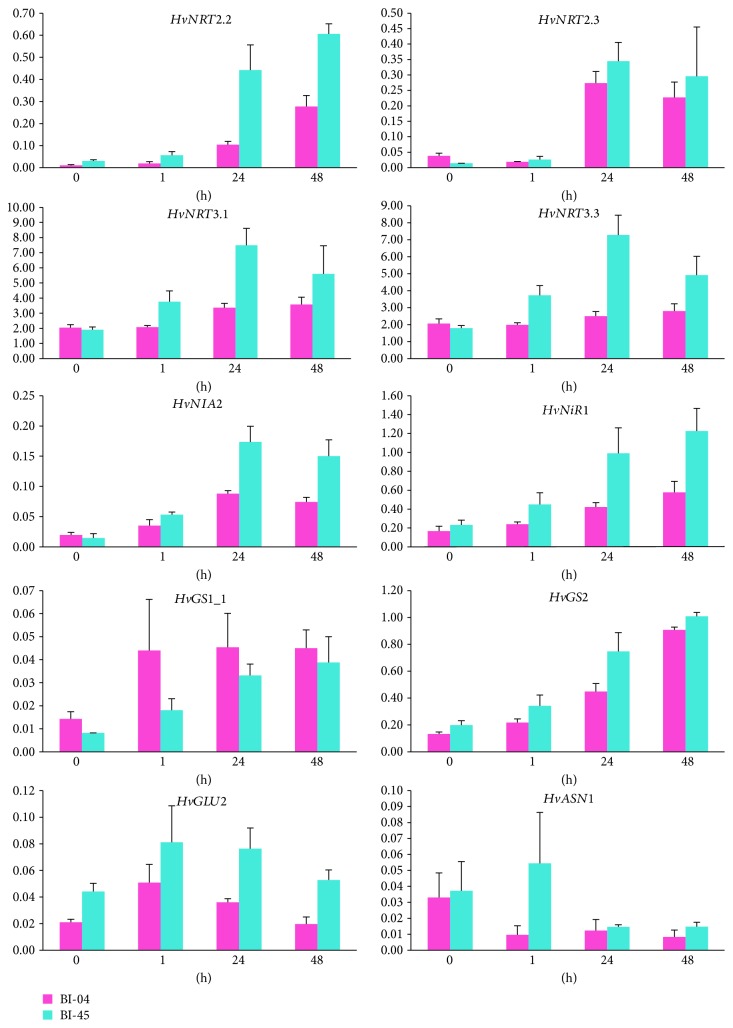
Differential expression of genes related to nitrogen metabolism in roots of the two barley cultivars. Roots were sampled at 0 h, 1 h, 24 h, and 48 h after low-nitrogen stress. Expression is represented as the normalized relative quantity (NRQ) of a target gene's expression with respect to the two reference genes: *HvActin* and *HvGAPDH*. Means and standard errors are shown from the analysis of three biological replicates.

The expression level of ten genes, comprising *HvNRT2.2*, *HvNRT2.3*, *HvNRT3.1*, *HvNRT3.3*, *HvNIA2*, *HvNiR1*, *HvGS1_1*, *HvGS2*, *HvGLU2*, and *HvASN1*, was analyzed in the roots of the two barley cultivars (Figure 3). ANOVA showed that the expression levels of *HvNRT2.2*, *HvNRT3.1*, *HvNRT3.3*, *HvNiR1*, *HvGS1_1*, *HvGS2*, and *HvGLU2* were significantly different in roots between the barley cultivars (*P* < 0.05). The expression levels of all genes except *HvASN1* showed significant differences in the roots at different time points (*P* < 0.05), and the expression of *HvNRT3.3* had a significant interaction between barley cultivars and time points (*P* < 0.05) (see Supplementary Table S2).

Multiple comparison analyses of gene expression showed that the expression levels of *HvNRT3.3* and *HvASN1* showed no significant changes in response to the low-nitrogen stress in BI-04 and the expression levels of *HvGLU2* and *HvASN1* showed no significant changes in BI-45 (see Supplementary Table S5). These results indicated that the gene regulation was more sensitive in the roots than in the shoots under low-nitrogen stress, especially in BI-45. For BI-04, *HvGS2* and *HvGLU2* were significantly induced at 1 h after low-nitrogen treatment (*P* < 0.05), and *HvNRT2.2*, *HvNRT3.1*, *HvNIA2*, *HvNiR1*, and *HvGS1_1* were significantly induced at 24 h after low-nitrogen treatment (*P* < 0.05), while *HvNRT2.3* expression was reduced at 1 h after low-nitrogen treatment and then induced at 24 h after low-nitrogen treatment. While in BI-45, *HvNRT3.3* and *HvNIA2* were significantly induced at 1 h after low-nitrogen treatment (*P* < 0.05), and *HvNRT2.2*, *HvNRT2.3*, *HvNRT3.1*, *HvNiR1*, *HvGS1_1*, and *HvGS2* were significantly induced at 24 h after low-nitrogen treatment (*P* < 0.05).

Comparing the two barley cultivars, *HvNRT2.2*, *HvNRT3.1*, *HvNiR1*, and *HvGS1_1* showed similar inductions, *HvNIA2* and *HvGS2* were induced in both cultivars but at different time points, while *HvNIA2* showed a rapid response in BI-45, and *HvGS2* showed a rapid response in BI-04. However, *HvNRT2.3*, *HvNRT3.3*, and *HvGLU2* showed different responses to low-nitrogen stress: *HvNRT2.3* was reduced at 1 h after low-nitrogen treatment and then induced from 24 h after low-nitrogen treatment in BI-04 while it was induced from 24 h after low-nitrogen treatment in BI-45; *HvNRT3.3* was only upregulated in BI-45, while *HvGLU2* was only induced in BI-04. These results suggested that there were different responses to low-nitrogen stress in terms of gene expression between BI-04 and BI-45, although there were no significant differences in root dry weight of each barley cultivar after low-nitrogen stress, and these different gene expressions might also contribute different effects of the two barley cultivars on low-nitrogen tolerance.

### 3.5. Different Gene Expression Patterns between Shoots and Roots of Two Barley Cultivars

The expression levels of *HvNRT3.1*, *HvNRT3.3*, *HvNIA2*, *HvNiR1*, *HvGS1_1*, *HvGS2*, *HvGLU2*, and *HvASN1* were compared between the shoots and the roots of the two barley cultivars. ANOVA showed that the expression levels of all genes had significant differences between the shoots and the roots of BI-04, all genes except *HvNRT3.3* and *HvASN1* had significant differences in expression among different time points, and only *HvGS2* had significant interactions between tissues and time points (*P* < 0.05) (see Supplementary Table S3). Meanwhile, all genes except *HvNIA2* and *HvGLU2* had significant differences in expression between shoots and roots in BI-45 (*P* < 0.05); all genes except *HvGLU2* and *HvASN1* had significant differences in expression among different time points (*P* < 0.05); and *HvNIA2* and *HvGS2* had significant interactions between tissues and time points (*P* < 0.05) (see Supplementary Table S4).

Comparing the gene regulation between shoots and roots of BI-04, *HvNRT3.1* and *HvNIA2* were only induced in the roots, and *HvGS1_1* and *HvGLU2* were induced in both the shoots and roots, but at different time points, while *HvASN1* expression was only reduced in shoots (see Supplementary Table S5). The gene expression levels of *HvNRT3.1*, *HvNRT3.3*, *HvNiR1*, *HvGS2*, and *HvGLU2* were different between the shoots and the roots at all time points, while *HvASN1* was different between shoots and roots only at 1 h after low-nitrogen treatment (see Supplementary Table S6).

For BI-45, *HvNRT3.1*, *HvNIA2*, *HvNiR1*, and *HvGS2* were induced in the roots, but no changes in the shoots, and *HvGS1_1* was induced in both the shoots and the roots at different time points of low-nitrogen treatment, while *HvASN1* expression was reduced such that it almost disappeared (only in the shoots) (see Supplementary Table S5). The gene expression levels of *HvNRT3.1*, *HvNRT3.3*, and *HvGS2* were different between shoots and roots at all time points, *HvNiR1* and *HvGLU2* were different at 0 h and 48 h after low-nitrogen treatment, the expression of *HvASN1* was different at 24 h and 48 h after low-nitrogen treatment, and *HvNIA2* and *HvGS1_1* were different only at 0 h after low-nitrogen treatment (see Supplementary Table S6).

These results indicated that gene expression, both in terms of regulation and expression levels, was very different between shoots and roots. Therefore, it was necessary to investigate gene expressions in shoots and roots separately.

## 4. Discussion

To identify the molecular mechanisms that are adopted by low-nitrogen-tolerant barley cultivars to adapt to low-nitrogen stress, we compared the differences in the expression levels of nitrogen metabolism-related genes between low-nitrogen-tolerant and low-nitrogen-sensitive barley cultivars. Kant et al. [19] compared gene expression levels in *Thellungiella halophila* with tolerance to low-nitrogen stress and *Arabidopsis* with sensitivity to low-nitrogen stress and suggested that *NR2*, *GS1*, *GS2*, *NRT2.1*, *NRT3.1*, and *NRT1.1* might be important in the adaptation to low-nitrogen stress in *Thellungiella.* In crops, Shi et al. [21] used two different rice cultivars to investigate their differences under low-nitrogen conditions and showed that *OsAMT1;1* and *OsNRT2;1* might play important roles in nitrogen acquisition. In trees, Luo et al. [20] also made a comparison of two contrasting *Populus* species and found that the strong responsiveness to limitation N supply by genes related to nitrogen metabolisms might be a good solution for acclimation to low-nitrogen stress in poplar.

In the present study, we compared the expression of genes related to nitrogen metabolism between a low-nitrogen-tolerant barley cultivar BI-04 and a low-nitrogen-sensitive barley cultivar BI-45 under low-nitrogen stress and found that *HvNiR1*, *HvGS2*, and *HvGLU2* were induced in shoots of BI-04, while *HvASN1* was reduced in both cultivars, and disappeared only in BI-45. In roots, we found that *HvGLU2* was only induced in BI-04, and *HvGS2* was induced from 1 h after low-nitrogen treatment in BI-04 while it was induced from 24 h after low-nitrogen treatment in BI-45. These results showed that *HvNiR1*, *HvGS2*, *HvGLU2*, and *HvASN1* might play important roles in low-nitrogen tolerance in BI-04, especially *HvGS2* and *HvGLU2* because of their induction in both shoots and roots of BI-04, and their stronger responses to low-nitrogen stress in the shoots of BI-04 than in BI-45, which might also be an important reason for BI-04's better adaptation to low-nitrogen stress.

The GS/GOGAT pathway is very important for primary nitrogen assimilation. This process changes inorganic nitrogen into organic nitrogen, which can then be directly absorbed by plants, and the inductions of *HvGS2* and *HvGLU2* in both shoots and roots might be one of the most important mechanisms underlying for the low-nitrogen tolerance of BI-04. A comparison of *Thellungiella halophila* with *Arabidopsis thaliana* showed that the former, as a low-nitrogen-tolerant species, had sustained the expression of *GS2* under low-nitrogen stress, while it was reduced in *Arabidopsis*, which grew poorly under N-limiting condition [19]. Furthermore, *GS2* was expressed in many tissues, including roots and leaves, while it was dominated in the leaves [30], and this phenomenon was also observed in our study. Additionally, Feraud et al. [31] showed that Fd-GOGAT was the most important enzyme in assimilation of photorespiratory and primary ammonium, especially in leaves. In our present study, the induction of *HvGS2* only in the shoots of BI-04 and the induction of *HvGLU2* in the shoots and roots of BI-04 might validate their predicted effects in the adaptation to low-nitrogen stress in barley.

Asparagine synthetase gene expression is dependent on nitrogen available and was reduced when nitrogen was limited [32, 33], and we also found that *HvASN1* was repressed only in shoots, indicating that the primary effects of nitrogen deficiency might appear initially in the shoots. Asparagine, which is synthesized by asparagine synthetase, is a key amino acid used to transport and store nitrogen in plants [7]. Overexpression of *ASN1* in *Arabidopsis* increased its tolerance to nitrogen-limiting stress [34]. Here, the lower repression of *HvASN1* in the shoots of BI-04 compared with that in BI-45 and the rapid induction of *HvASN1* in the roots of BI-04 might lead to better adaptation to low-nitrogen stress.

In addition, transgenic *Arabidopsis* with the spinach nitrite reductase gene showed an improvement in NO_2_ assimilation in shoots [35]. Therefore, the induction of *HvNiR1* in the shoots of BI-04 might have some effects on incorporating NO_2_ in the atmosphere to relieve low-nitrogen stress.

## 5. Conclusion

In this study, two barley cultivars with different adaptations to low-nitrogen stress were used to investigate the molecular mechanism of barley's response to low-nitrogen tolerance. Our results showed that the increased expression levels of *HvNiR1*, *HvGS2*, and *HvGLU2*, the less decreased expression of *HvASN1* in shoots under low-nitrogen stress, and the increased expression of *HvGLU2* and the rapid response of *HvGS2* in roots under low-nitrogen stress, could benefit adaptation to low-nitrogen stress in barley. The expressions of these genes will be preferentially detected to identify low-nitrogen-tolerant barley germplasms in the future. We also provided two important barley cultivars for exploring the in-depth molecular mechanism of low-nitrogen tolerance: one cultivar could maintain its biomass under early nitrogen deficiency, while the other could not. Furthermore, we also emphasized the importance of detecting gene expression in different barley tissues to completely reveal the mechanism of adaptation to low-nitrogen stress in barley.

## Figures and Tables

**Table 1:** Primers for qRT-PCR.

Gene name	Accession number	Prime sequences (5′ to 3′)		Amplicon (bp)	Origin
*HvNRT2.2*	U34290.1	Forward	TCCTTCTTCACCTGCTTCGT	80	This study
		Reverse	TTGGCGAGGTTTAGGTTGTC		
*HvNRT2.3*	AF091115.1	Forward	ATGGCGTATTGCCTACTTCG	90	
		Reverse	TTCCCATCAGGGAGATCTTG		
*HvNRT3.1*	AY253448.1	Forward	GAACGTGAAGGTGAGCCTCT	96	
		Reverse	TGGCAGGTCTTGTCCTTCTT		
*HvNRT3.3*	AY253450.1	Forward	AAGGACGCCGACTACAAGAA	131	
		Reverse	TGCTGGGTGATCTTGAACTG		
*HvNIA2*	X57845.1	Forward	TGGCAAGAAGATCACACGAG	120	
		Reverse	CAGAAGCACCAGCACCAGTA		
*HvNiR1*	S78730.1	Forward	CTCACCGGGGTGTACAAGAA	114	
		Reverse	CTCCTCGTCCTCCTCCCTCT		
*HvGS1_1*	X69087.1	Forward	GTTCAGGGAGGGAAACAACA	112	
		Reverse	ATCGGGGTTGCTAAGGATCT		
*HvGS2*	X53580.1	Forward	ATAGCCGCATATGGTGAAGG	106	
		Reverse	GAATAGAGCAGCCACGGTTC		
*HvGLU2*	S58774.1	Forward	ACCAATGAGGTTGCTTGGAC	85	
		Reverse	TATTGTGGCTTCCCTTGACC		
*HvASN1*	AF307145.1	Forward	AAGGAGGGAGGCTTCAAGAG	146	
		Reverse	AGAACACCGAATGGAACGTC		

*HvActin*	AY145451.1	Forward	TGAGGCGCAGTCCAAGAGA	81	Chen et al. [26]
		Reverse	TCCATGTCATCCCAGTTGCTTA		
*HvGAPDH*	X60343.1	Forward	ACAGTTCACGGCCATTGGA	102	
		Reverse	AGGGTTCCTGACGCCAAAG		

## Abbreviations

NRT1: Low-affinity nitrate transport
NRT2: High-affinity nitrate transport
AMT: Ammonium transporters
NR: Nitrate reductase
NiR: Nitrite reductase
GS: Glutamine synthetase
GOGAT: Glutamate synthase
AS: Asparagine synthetase
NRQ: Normalized relative quantity.

## Supplementary Materials

Supplementary Materials Table S1: two-way ANOVA of Ct value of each gene expression at different time points after low-nitrogen stress in shoots of the two barley cultivars. Table S2: two-way ANOVA of Ct value of each gene expression at different time points after low-nitrogen stress in roots of the two barley cultivars. Table S3: two-way ANOVA of Ct value of each gene expression at different time points after low-nitrogen stress between shoots and roots of BI-04. Table S4: two-way ANOVA of Ct value of each gene expression at different time points after low-nitrogen stress between shoots and roots of BI-45. Table S5: Ct value of gene expression at different time points after low-nitrogen stress and the significant difference analysis in shoots and roots of the two barley cultivars. Table S6: Ct value of gene expression at different time points and significant difference analysis between shoots and roots of the two barley cultivars.
