# Supplementary material for: Expression Analysis of Nitrogen Metabolism-Related Genes Reveals Differences in Adaptation to Low-Nitrogen Stress between Two Different Barley Cultivars at Seedling Stage
Source: Int J Genomics. 2018 Jun 20;2018:8152860. doi: 10.1155/2018/8152860 (PMC6031091; doi:10.1155/2018/8152860)
Supplement: Supplementary Materials — Table S1: two-way ANOVA of Ct value of each gene expression at different time points after low-nitrogen stress in shoots of the two barley cultivars. Table S2: two-way ANOVA of Ct value of each gene expression at different time points after low-nitrogen stress in roots of the two barley cultivars. Table S3: two-way ANOVA of Ct value of each gene expression at different time points after low-nitrogen stress between shoots and roots of BI-04. Table S4: two-way ANOVA of Ct value of each gene expression at different time points after low-nitrogen stress between shoots and roots of BI-45. Table S5: Ct value of gene expression at different time points after low-nitrogen stress and the significant difference analysis in shoots and roots of the two barley cultivars. Table S6: Ct value of gene expression at different time points and significant difference analysis between shoots and roots of the two barley cultivars. [file 8152860.f1.pdf]

Supplementary materials

Table S1. Two-way ANOVA of Ct' value of each gene expression at different time points after low-nitrogen stress in shoots of the two barley cultivars

Table S2. Two-way ANOVA of Ct' value of each gene expressionat different time points after low-nitrogen stress in roots of the two barley cultivars

Table S3. Two-way ANOVA of Ct' value of each gene expressionat different time points after low-nitrogen stress between shoots and roots of BI-04

Table S4. Two-way ANOVA of Ct' value of each gene expressionat different time points after low-nitrogen stress between shoots and roots of BI-45

Table S5. Ct' value of gene expression at different time points after low-nitrogen stress and the significant difference analysis in shoots and roots of the two barley cultivars

Table S6. Ct' value of gene expression at different time points and significant difference analysis between shoots and roots of the two barley cultivars

Table S1. Two-way ANOVA of Ct' value of each gene expression at different time points after low-nitrogen stress in shoots of the two barley cultivars

Source	Gene	SS	df	MS	F	Sig.
Cultivar	*HvNRT3.1*	0.216	1	0.216	0.379	0.551
	HvNRT3.3	0.038	1	0.038	0.040	0.846
	HvNIA2	0.678	1	0.678	1.217	0.294
	HvNiR1	1.144	1	1.144	1.384	0.259
	HvGS1_1	1.252E-05	1	1.252E-05	0.000	0.992
	HvGS2	1.786	1	1.786	5.373	0.034
	HvGLU2	0.818	1	0.818	0.459	0.508
	HvASN1	0.330	1	0.330	1.214	0.303
Time Point	*HvNRT3.1*	4.884	3	1.628	2.852	0.086
	HvNRT3.3	2.543	3	0.848	0.893	0.478
	HvNIA2	2.591	3	0.864	1.549	0.257
	HvNiR1	6.916	3	2.305	2.789	0.079
	HvGS1_1	5.197	3	1.732	16.334	0.001
	HvGS2	1.185	3	0.395	1.188	0.346
	HvGLU2	3.451	3	1.150	0.646	0.597
	HvASN1	7.391	3	2.464	9.070	0.006
Cultivar × Time Point	*HvNRT3.1*	1.499	3	0.500	0.875	0.483
	HvNRT3.3	0.216	2	0.108	0.114	0.894
	HvNIA2	0.849	3	0.283	0.507	0.685
	HvNiR1	1.363	3	0.454	0.549	0.657
	HvGS1_1	1.248	3	0.416	3.922	0.048
	HvGS2	2.286	3	0.762	2.293	0.117
	HvGLU2	13.271	3	4.424	2.483	0.098
	HvASN1	0.012	1	0.012	0.043	0.841
Total	*HvNRT3.1*	338.511	19			
	HvNRT3.3	285.484	17			
	HvNIA2	274.413	19			
	HvNiR1	45.332	22			
	HvGS1_1	330.869	17			
	HvGS2	367.074	24			
	HvGLU2	264.552	24			
	HvASN1	197.599	14			

Table S2. Two-way ANOVA of Ct’ value of each gene expression at different time points after low-nitrogen stress in roots of the two barley cultivars

Souce	Gene	SS	df	MS	F	Sig.
Cultivar	*HvNRT2.2*	15.224	1	15.224	35.243	0.000
	HvNRT2.3	0.107	1	0.107	0.258	0.619
	HvNRT3.1	2.086	1	2.086	13.396	0.002
	HvNRT3.3	3.366	1	3.366	26.148	0.000
	HvNIA2	1.152	1	1.152	2.392	0.143
	HvNiR1	4.625	1	4.625	14.021	0.002
	HvGS1_1	2.241	1	2.241	4.824	0.045
	HvGS2	1.480	1	1.480	13.763	0.002
	HvGLU2	6.497	1	6.497	26.644	0.000
	HvASN1	4.484	1	4.484	3.096	0.109
Time Point	*HvNRT2.2*	84.236	3	28.079	65.001	0.000
	HvNRT2.3	67.433	3	22.478	54.418	0.000
	HvNRT3.1	6.630	3	2.210	14.190	0.000
	HvNRT3.3	4.575	3	1.525	11.847	0.000
	HvNIA2	36.998	3	12.333	25.603	0.000
	HvNiR1	14.831	3	4.944	14.988	0.000
	HvGS1_1	11.715	3	3.905	8.408	0.002
	HvGS2	23.116	3	7.705	71.633	0.000
	HvGLU2	4.255	3	1.418	5.817	0.008
	HvASN1	5.916	3	1.972	1.362	0.310
Cultivar × Time Point	*HvNRT2.2*	0.531	3	0.177	0.410	0.748
	HvNRT2.3	2.369	3	0.790	1.912	0.174
	HvNRT3.1	1.229	3	0.410	2.629	0.086
	HvNRT3.3	2.243	3	0.748	5.808	0.007
	HvNIA2	3.246	3	1.082	2.246	0.125
	HvNiR1	0.282	3	0.094	0.285	0.836
	HvGS1_1	0.641	3	0.214	0.460	0.715
	HvGS2	0.272	3	0.091	0.844	0.491
	HvGLU2	0.421	3	0.140	0.576	0.640
	HvASN1	3.971	3	1.324	0.914	0.469
Total	*HvNRT2.2*	411.789	23			
	HvNRT2.3	351.726	22			
	HvNRT3.1	82.016	24			
	HvNRT3.3	70.878	24			
	HvNIA2	466.910	23			
	HvNiR1	61.300	23			
	HvGS1_1	636.533	22			
	HvGS2	68.875	23			
	HvGLU2	511.137	23			
	HvASN1	656.328	18			

Table S3. Two-way ANOVA of Ct’ value of each gene expressionat different time points after low-nitrogen stress between shoots and roots of BI-04

Source	Gene	SS	df	MS	F	Sig.
Tissue	*HvNRT3.1*	175.118	1	175.118	1034.642	0.000
	HvNRT3.3	141.376	1	141.376	291.690	0.000
	HvNIA2	1.734	1	1.734	5.858	0.030
	HvNiR1	35.283	1	35.283	93.812	0.000
	HvGS1_1	1.975	1	1.975	6.151	0.029
	HvGS2	162.114	1	162.114	1759.644	0.000
	HvGLU2	32.016	1	32.016	134.911	0.000
	HvASN1	26.347	1	26.347	30.617	0.000
Time Point	*HvNRT3.1*	4.370	3	1.457	8.607	0.002
	HvNRT3.3	1.701	3	0.567	1.170	0.359
	HvNIA2	7.689	3	2.563	8.658	0.002
	HvNiR1	13.085	3	4.362	11.597	0.000
	HvGS1_1	6.404	3	2.135	6.648	0.007
	HvGS2	10.938	3	3.646	39.573	0.000
	HvGLU2	6.933	3	2.311	9.738	0.001
	HvASN1	4.284	3	1.428	1.659	0.233
Tissue× Time Point	*HvNRT3.1*	0.812	3	0.271	1.599	0.234
	HvNRT3.3	0.840	3	0.280	0.578	0.640
	HvNIA2	2.812	3	0.937	3.167	0.058
	HvNiR1	0.216	3	0.072	0.191	0.901
	HvGS1_1	0.520	3	0.173	0.540	0.664
	HvGS2	4.588	3	1.529	16.599	0.000
	HvGLU2	1.569	3	0.523	2.204	0.127
	HvASN1	8.815	3	2.938	3.414	0.057
Total	*HvNRT3.1*	210.110	22			
	HvNRT3.3	177.900	21			
	HvNIA2	405.440	22			
	HvNiR1	61.898	23			
	HvGS1_1	461.833	20			
	HvGS2	202.271	24			
	HvGLU2	425.956	24			
	HvASN1	541.360	19			

Table S4. Two-way ANOVA of Ct’ value of each gene expressionat different time points after low-nitrogen stress between shoots and roots of BI-45

Source	Gene	SS	df	MS	F	Sig.
Tissue	*HvNRT3.1*	188.150	1	188.150	382.090	0.000
	HvNRT3.3	162.322	1	162.322	402.346	0.000
	HvNIA2	1.218	1	1.218	1.586	0.232
	HvNiR1	22.584	1	22.584	29.060	0.000
	HvGS1_1	7.624	1	7.624	23.275	0.001
	HvGS2	154.557	1	154.557	424.735	0.000
	HvGLU2	4.255	1	4.255	2.250	0.154
	HvASN1	8.528	1	8.528	8.303	0.024
Time Point	*HvNRT3.1*	8.222	3	2.741	5.566	0.011
	HvNRT3.3	7.107	3	2.369	5.872	0.009
	HvNIA2	9.236	3	3.079	4.010	0.034
	HvNiR1	9.035	3	3.012	3.875	0.033
	HvGS1_1	10.090	3	3.363	10.268	0.002
	HvGS2	6.822	3	2.274	6.249	0.006
	HvGLU2	2.742	3	0.914	0.483	0.699
	HvASN1	3.819	3	1.273	1.239	0.365
Tissue × Time Point	*HvNRT3.1*	0.327	3	0.109	0.221	0.880
	HvNRT3.3	0.045	2	0.023	0.056	0.946
	HvNIA2	17.133	3	5.711	7.438	0.004
	HvNiR1	1.668	3	0.556	0.715	0.559
	HvGS1_1	0.729	3	0.243	0.742	0.549
	HvGS2	4.722	3	1.574	4.325	0.022
	HvGLU2	9.881	3	3.294	1.742	0.201
	HvASN1	0.062	1	0.062	0.060	0.813
Total	*HvNRT3.1*	210.417	21			
	HvNRT3.3	178.461	20			
	HvNIA2	335.883	20			
	HvNiR1	44.733	22			
	HvGS1_1	505.568	19			
	HvGS2	233.678	23			
	HvGLU2	349.733	23			
	HvASN1	312.567	13			

Table S5. Ct' value of gene expression at different time points after low-nitrogen stress and the significant difference analysis in shoots and roots of the two barley cultivars

Cultivar	tissue	gene	Time			
			0h	1h	24h	48h
BI-04	shoots	*HvNRT3.1*	2.5242	3.2238	4.0189	3.7157
		HvNRT3.3	2.5150	2.7862	1.8412	4.2263
		HvNIA2	3.9454	4.4565	3.6706	3.4719
		HvNiR1	0.3571a	0.3204ab	-1.5360c	-1.4461bc
		HvGS1_1	5.1318a	4.2449b	3.8322b	4.2746b
		HvGS2	-2.8683a	-3.8355b	-3.9644b	-3.6570b
		HvGLU2	3.9071a	2.3470b	2.3832b	2.6935b
		HvASN1	3.5239ab	2.9935a	4.8000bc	5.0842c
	roots	*HvNRT2.2*	6.7723a	5.9227a	3.2888b	1.8972b
		HvNRT2.3	4.7880a	5.7318b	1.8953c	2.2047c
		HvNRT3.1	-1.0198a	-1.0565a	-1.7425b	-1.8126b
		HvNRT3.3	-1.0148	-0.9863	-1.3063	-1.4467
		HvNIA2	5.7547a	4.9258a	3.5062b	3.7656b
		HvNiR1	2.7222a	2.0690ab	1.2638bc	0.8654c
		HvGS1_1	6.2142a	4.7159ab	4.6027b	4.5166b
		HvGS2	2.9248a	2.2228b	1.1785c	0.1406d
		HvGLU2	5.5853a	4.4208b	4.7993ab	5.7652a
		HvASN1	5.2107	7.0003	6.6396	7.1538
BI-45	shoots	*HvNRT3.1*	2.9189	4.3348	3.6058	3.5165
		HvNRT3.3	ND	4.3781	3.5497	3.9017
		HvNIA2	4.0654	4.1348	3.5576	3.5701
		HvNiR1	-0.8519	-0.8786	-1.3155	-1.8846
		HvGS1_1	5.4732a	4.2707b	4.3229ab	3.4097b
		HvGS2	-4.1740	-3.9364	-3.7611	-4.6361
		HvGLU2	2.3878	4.1788	4.2006	2.0410
		HvASN1	3.0834	2.6925	ND	ND
	roots	*HvNRT2.2*	5.0655a	4.2191a	1.2984b	0.7303b
		HvNRT2.3	6.1106a	5.3524a	1.5773b	2.1455b
		HvNRT3.1	-0.9174a	-1.8583ab	-2.8721c	-2.3423bc
		HvNRT3.3	-0.8259a	-1.8644b	-2.8286c	-2.2312bc
		HvNIA2	6.5658a	4.2340b	2.5597b	2.7860b
		HvNiR1	2.1342a	1.2808ab	0.1203bc	-0.2347c
		HvGS1_1	6.9357a	5.9080ab	4.9494b	4.8490b
		HvGS2	2.3697a	1.5854a	0.4742b	-0.0106b
		HvGLU2	4.5295	3.7097	3.7679	4.2733
		HvASN1	5.2287	4.5014	6.1026	6.1170

Different letters represent significant differences of the related gene expression among different time points at 0.05 level in shoots and root respectively of BI-04 and Bi-45. ND indicates that expression was not detectable.

Table S6. Ct' value of gene expression at different time points and significant difference analysis between shoots and roots of the two barley cultivars

Cultivar	Gene	Tissue	Time			
			0h	1h	24h	48h
BI-04	*HvNRT3.1*	shoots	2.5242a	3.2238a	4.0189a	3.7157a
		roots	-1.0198b	-1.0565b	-1.7425b	-1.8126b
	HvNRT3.3	shoots	2.5150a	2.7862a	1.8412a	4.2263a
		roots	-1.0148b	-0.9863b	-1.3063b	-1.4467b
	HvNIA2	shoots	3.9454	4.4565	3.6706	3.4719
		roots	5.7547	4.9258	3.5062	3.7656
	HvNiR1	shoots	0.3571a	0.3204a	-1.5360a	-1.4461a
		roots	2.7222b	2.0690b	1.2638b	0.8654b
	HvGS1_1	shoots	5.1318	4.2449	3.8322	4.2746
		roots	6.2142	4.7159	4.6027	4.5166
	HvGS2	shoots	-2.8683a	-3.8355a	-3.9644a	-3.6570a
		roots	2.9248b	2.2228b	1.1785b	0.1406b
	HvGLU2	shoots	3.9071a	2.3470a	2.3832a	2.6935a
		roots	5.5853b	4.4208b	4.7993b	5.7652b
	HvASN1	shoots	3.5239	2.9935a	4.8000	5.0842
		roots	5.2107	7.0003b	6.6396	7.1538
BI-45	*HvNRT3.1*	shoots	2.9189a	4.3348a	3.6058a	3.5165a
		roots	-0.9174b	-1.8583b	-2.8721b	-2.3423b
	HvNRT3.3	shoots	ND	4.3781a	3.5497a	3.9017a
		roots	-0.8259	-1.8644b	-2.8286b	-2.2312b
	HvNIA2	shoots	4.0654a	4.1348	3.5576	3.5701
		roots	6.5658b	4.2340	2.5597	2.7860
	HvNiR1	shoots	-0.8519a	-0.8786	-1.3155	-1.8846a
		roots	2.1342b	1.2808	0.1203	-0.2347b
	HvGS1_1	shoots	5.4732a	4.2707	4.3229	3.4097
		roots	6.9357b	5.9080	4.9494	4.8490
	HvGS2	shoots	-4.1740a	-3.9364a	-3.7611a	-4.6361a
		roots	2.3697b	1.5854b	0.4742b	-0.0106b
	HvGLU2	shoots	2.3878a	4.1788	4.2006	2.0410a
		roots	4.5295b	3.7097	3.7679	4.2733b
	HvASN1	shoots	3.0834	2.6925	ND	ND
		roots	5.2287	4.5014	6.1026	6.1170

Different letters represent significant differences of the related gene expression between shoots and roots at 0.05 level in BI-04 and BI-45 respectively. ND indicates that the expression was not detectable.
